# Hydroxyapatite Coated Iron Oxide Nanoparticles: A Promising Nanomaterial for Magnetic Hyperthermia Cancer Treatment

**DOI:** 10.3390/nano7120426

**Published:** 2017-12-04

**Authors:** Sudip Mondal, Panchanathan Manivasagan, Subramaniyan Bharathiraja, Madhappan Santha Moorthy, Van Tu Nguyen, Hye Hyun Kim, Seung Yun Nam, Kang Dae Lee, Junghwan Oh

**Affiliations:** 1 Marine-Integrated Bionics Research Center, Pukyong National University, Busan 48513, Korea; mailsudipmondal@gmail.com (S.M.); manimaribtech@gmail.com (P.M.); sbrbtc@gmail.com (S.B.); santham83@gmail.com (M.S.M.); Hye.H.Kim@colorado.edu (H.H.K.); synam@pknu.ac.kr (S.Y.N.); 2 Interdisciplinary Program of Biomedical Mechanical and Electrical Engineering, Pukyong National University, Busan 48513, Korea; nguyen.vantu91@gmail.com; 3 Department of Biomedical Engineering and Center for Marine-Integrated Biotechnology (BK21 Plus), Pukyong National University, Busan 48513, Korea; 4 Department of Otolaryngology—Head and Neck Surgery, Kosin University College of Medicine, Busan 602-702, Korea; kdlee59@gmail.com

**Keywords:** hydroxyapatite, iron oxide, hydroxyapatite coated iron oxide, magnetic hyperthermia, cancer therapy

## Abstract

Targeting cancer cells without injuring normal cells is the prime objective in treatment of cancer. In this present study, solvothermal and wet chemical precipitation techniques were employed to synthesize iron oxide (IO), hydroxyapatite (HAp), and hydroxyapatite coated iron oxide (IO-HAp) nanoparticles for magnetic hyperthermia mediated cancer therapy. The synthesized well dispersed spherical IO-HAp nanoparticles, magnetite, and apatite phases were confirmed by X-ray diffraction (XRD), Fourier-transform infrared spectroscopy (FTIR) and Field emission transmission electron microscopy (FETEM) with Energy Dispersive X-ray spectroscopy (EDS). The non-toxic behavior of synthesized IO-HAp nanoparticles was confirmed by cytotoxicity assay (Trypan blue and MTT assay). The synthesized nanoparticles revealed a remarkable magnetic saturation of 83.2 emu/g for IO and 40.6 emu/g for IO-HAp nanoparticles in presence of 15,000 Oe (1.5 T) magnetic field at room temperature (300 K). The magnetic hyperthermia study that was performed with IO-HAp nanoparticles showed an excellent hyperthermia effect (SAR value 85 W/g) over MG-63 osteosarcoma cells. The in vitro hyperthermia temperature (~45 °C) was reached within 3 min, which shows a very high efficiency and kills nearly all of the experimental MG-63 osteosarcoma cells within 30 min exposure. These results could potentially open new perceptions for biomaterials that are aimed for anti-cancer therapies based on magnetic hyperthermia.

## 1. Introduction

Nanotechnology advanced the functional characteristics of nanoparticles for biomedical applications. The advances to treat diseases, such as cancer, enhanced bioimaging property, and controlled drug releasing ability make them very significant for biomedical research [1]. The prime difficulties are presently related to the systematic administration and biodistribution of drugs, inadequate drug concentration at the clinical site, toxicity due to nonspecific targeting, and high drug concentration related issues. Magnetic drug targeting solves many of these problems [2]. Since the last decade, magnetic ceramic composite has been one of the most interesting topics for researchers to study its application in the biomedical field [3]. To date, many research articles are focusing on nanomaterials in magnetic hyperthermia study, but for real medicinal application, it confronts many unavoidable complications, among which the early toxicity is the most common one.

**Scheme 1:**
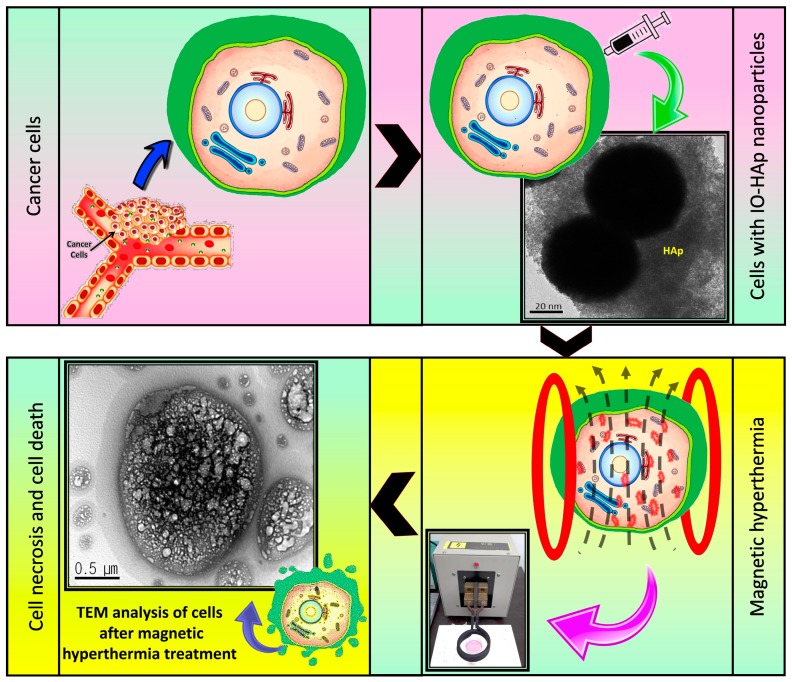
Magnetic hydroxyapatite (IO-HAp) mediated hyperthermia study to treat cancer.

Magnetic hyperthermia deals with the magnetic materials to generate heat under an alternating magnetic field. Magnetic hyperthermia principle depends upon the capability of magnetic moment oscillation to magnetic materials, results in generation of heat via converting magnetic energy, which affects the surrounding tissues to damage the targeted cells [4]. Superparamagnetic (single domain magnetic materials without magnetic memory) nanoparticles are the most promising nanomaterials for magnetic hyperthermia treatment [5]. Iron oxide nanoparticles generate heat in response to the external magnetic field, and after removal of the magnetic field, does not retain any magnetism [6]. Gilchrist et al. (1957) first proposed two different approaches for iron oxide-mediated tumor heating/killing [7]. Magnetic hyperthermia is the first technique that is associated with heat generation (45–47 °C) by nanoparticles. Presently, magnetic hyperthermia is well adopted along with radiotherapy or chemotherapy for different cancer treatment [8]. Magnetic thermoablation is another technique that generates the heat by increasing the temperature to 43–55 °C with strong toxic effect on cells [9,10]. Lubbe et al. 2001, reported the first clinical magnetic drug targeting trials in humans, with epirubicin antibiotic-loaded ferrofluid (average particle size of 100 nm for treating solid tumors) [11]. Fe_3_O_4_ nanoparticles, with its superparamagnetic properties, have numerous biomedical applications. However, the direct use of only Fe_3_O_4_ nanoparticles is strongly discouraged due to their biofouling in blood plasma, which causes agglomeration and segregation by the reticular endothelial system [12]. Different study reports propose that the direct contact of Fe_3_O_4_ nanoparticles with cells induces detrimental effects due to the presence of iron as a redox center, which causes oxidative stress by releasing reactive oxygen species (ROS), such as hydroxyl, superoxide, and hydrogen peroxide radicals. Therefore, it is very important to modify the Fe_3_O_4_ nanoparticles’ surface to diminish agglomeration and biofouling in biological conditions. There are numerous approaches employed for surface modification of Fe_3_O_4_ nanoparticles that involve employing ceramics, polymers, composites, etc. Among the polymers, polyethylene glycol, dextran, polyvinyl alcohol, polyvinylpyrrolidone, starch, etc. are well reported [12]. However, the polymer coating can easily dissolve due to the changes of pH, temperature, chemical or enzymatic reactions, solubility in body fluids, etc. [12]. An inorganic matrix could be an effective coating material for tailoring well-dispersed, ultrafine, uniform nanostructures [13]. Although many inorganic materials have been studied for this purpose, hydroxyapatite (HAp) [Ca_5_(PO_4_)_3_(OH)], the most suitable inorganic coating material [14]. HAp is extensively used as biomaterial due to its excellent biocompatibility, bioactivity, and osteoconductivity [15]. Many researchers worldwide reported a range of HAp or HAp-supported magnetic materials for different biomedical applications, including hyperthermia-based cancer treatment [16,17,18,19,20,21,22]. Murakami et al. synthesized porous Fe_3_O_4_-HAp by employing the hydrothermal synthesis technique, and showed that the rod-shaped HAp particles hold 30% Fe_3_O_4_ in their composite cage structure [19]. Donadel et al. performed the spray–drying (Büchi B-191 dryer) to synthesize a spherical core/shell Fe_3_O_4_ coated HAp structure [20]. Tampieri et al. synthesized doped Fe^2+^/Fe^3+^ on HAp nanostructures for magnetic hyperthermia study [21]. Until now, most reports show the lack of suitable synthetic strategies with high reproducibility. Moreover, the phase change of Fe_3_O_4_ nanoparticles during surface modification causes changes in magnetic saturation, which have a great impact on the hyperthermia study. The present study reports a facile synthetic procedure of magnetic HAp by two-step hydrothermal and wet chemical precipitation techniques. First, a controlled hydrothermal synthesis procedure was employed to synthesize Fe_3_O_4_ nanoparticles, and was followed by coating with HAp. The synthesized IO, HAp, and IO-HAp nanoparticles cytotoxicity study were performed with MG-63 osteosarcoma cell lines. The IO-HAp nanoparticles further studied for in vitro hyperthermia assessment. The promising results encourage the future application of IO-HAp nanomaterials as a nano heater for magnetic hyperthermia mediated cancer therapy (Scheme 1).

## 2. Experiment

### 2.1. Chemical Reagents

The following chemical reagents were used in this study: Iron (III) chloride hexahydrate (FeCl_3_·6H_2_O) (Sigma Aldrich, St. Louis, MO, USA), ethylene glycol (Samchun Chemicals, Seoul, Korea), trisodium citrate (DC Chemicals, Seoul, Korea), calcium nitrate tetrahydrate [Ca(NO_3_)_2_·4H_2_O] (Samchun Chemicals, Seoul, Korea), diammonium hydrogen phosphate [(NH_4_)_2_HPO_4_], sodium acetate, and ammonium hydroxide (NH_4_OH) (28%) (Sigma Aldrich, St. Louis, MO, USA). Reagents were used without any further purification.

### 2.2. Synthesis of HAp Nanoparticles

HAp nanoparticles were synthesized by chemical precipitation using Ca(NO_3_)_2_·4H_2_O and (NH_4_)_2_HPO_4_ as precursors [23]. A suspension of 0.24 M Ca(NO_3_)_2_·4H_2_O (23.6 g Ca(NO_3_)_2_·4H_2_O in 350 mL distilled water) was stirred while maintaining the temperature at 40 °C. Next, the solution of 0.29 M (NH_4_)_2_HPO_4_ (7.9 g (NH_4_)_2_HPO_4_ in 250 mL distilled water) was gently added to the Ca(NO_3_)_2_·4H_2_O solution. During experimentation, the pH was adjusted to 11 by using an NH_4_OH solution. The chemical reaction is expressed as:

10 Ca(NO_3_)_2_·4H_2_O + 6 (NH_4_)_2_HPO_4_ + 8 NH_4_OH → Ca_10_(PO_4_)_6_(OH)_2_ + 20 NH_4_NO_3_ + 20 H_2_O

The synthesized HAp nanoparticles were dried at 70 °C and calcined at 600 °C for one hour in the air atmosphere.

### 2.3. Synthesis of Magnetic Fe_3_O_4_ Nanoparticles

To synthesize magnetic Fe_3_O_4_ nanoparticles, the solvothermal method was employed [24]. A 0.6 M FeCl_3_·6H_2_O solution was prepared by dissolving 1.621 g of ferric chloride salt in 50 mL of ethylene glycol and stirred under nitrogen flow for 30 min. Next, 1.3 M of sodium acetate was added under stirring. Finally, 0.5 g of trisodium citrate was added, followed by continuous stirring for 3 h at room temperature. The final mixture was transferred to an autoclave chamber (100-mL capacity Teflon-lined stainless steel) and heated at 190 °C for 10 h. Finally, after cooling down the autoclave to room temperature, the obtained dark black precipitate was washed several times with deionized (DI) water and ethanol and dried at 65 °C for 12 h, followed by storing under a nitrogen atmosphere. The synthesized Fe_3_O_4_ nanoparticles were highly hydrophilic and could be easily dispersed in water.

### 2.4. Synthesis of Fe_3_O_4_-HAp (IO-HAp) Nanocomposites

**Figure 1:**
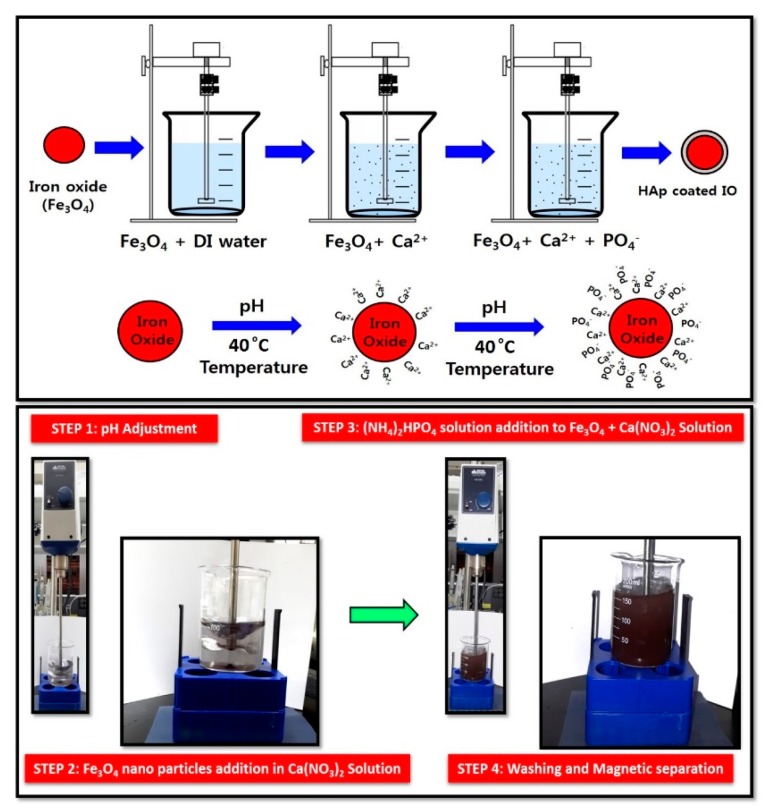
Synthesis of hydroxyapatite coated iron oxide (IO-HAp) nanoparticles.

The IO-HAp nanocomposites were synthesized by wet chemical precipitation technique (Figure 1). The synthesized iron oxide (IO) nanoparticles were well dispersed in a 0.06 M calcium nitrate tetrahydrate 70 mL solution, and were stirred vigorously with a mechanical stirrer. During this the procedure, Ca^2+^ ions initiate its nucleation over IO surface due to its favorable zeta potential. Next, 50 mL of a 0.07 M di-ammonium hydrogen phosphate solution was gently added dropwise to the IO-containing solution. The next step followed by nucleation of PO_4_^3−^ ions deposition over Ca^2+^ to form HAp crystals. The whole experimental study was performed at 40 °C temperature with pH 9.0. Soon after addition of the di-ammonium hydrogen phosphate, the clear solution became cloudy due to the formation of the HAp coating over the IO nanoparticles. The solution was continuously agitated with a mechanical stirrer for 5 h, and was finally washed with deionized water. The nanoparticles were dried at 80 °C for 6 h, and were finally kept in a vial for further application.

### 2.5. Characterization

A powder X-ray diffraction (XRD) study was performed by a Bruker AXN XRD analyzer, (Karlsruhe, Germany). Fourier transform infrared spectra was measured by using JASCO FTIR 4100 (Tokyo, Japan), with wavenumber range of 450 to 4000 cm^−1^. The Fourier-transform infrared spectroscopy (FTIR) pellets were prepared by mixing the samples with Potassium bromide (KBr). Thermogravimetric analysis was performed by Perkin-Elmer Pyris Diamond at a heating rate of 10 °C/min in a nitrogen atmosphere. Field emission transmission electron microscopy (FETEM) was performed using FETEM, JEOL 2010 (Tokyo, Japan), instrument. The average hydrodynamic size of nanoparticles was determined by dynamic light scattering (DLS), using particle size analyzer (Beckman Coulter, Brea, CA, USA). The magnetic saturation study was performed by superconducting quantum interference device magnetometer (Quantum design, MPMS XL).

### 2.6. Cell Culture

MG-63 osteosarcoma cells (Korea Cell Line Bank, Seoul, Korea) were used and cultured in Dulbecco’s Modified Eagle Medium (DMEM, Thermo Scientific, Waltham, MA, USA), supplemented with 10% fetal bovine serum and 100 U mL^−1^ Penicillin and 100 µg mL^−1^ Streptomycin (Thermo Fischer Scientific, Waltham, MA, USA). The cells were incubated at 37 °C temperature in 5% CO_2_ atmosphere. The growth medium was changed after each 24 h of incubation time interval.

### 2.7. Cell Viability Assay

#### 2.7.1. MTT Assay

Before hyperthermia study, the cytotoxic effect of the synthesized nanoparticles was assessed. MG-63 cells were seeded at a density of 10^4^ cells/well in a 96-well plate with 100 μL of DMEM for 24 h. Different concentrations of IO-HAp, IO and HAp nanoparticles (20, 40, 60, 80, 100, or 120 µg/mL) were then added to the culture medium. 3-(4,5-dimethylthiazol-2-yl)2,5-diphenyltetrazolium bromide (MTT) and Trypan blue cytotoxicity assays were performed to examine the in vitro cytotoxicity of IO-HAp. The control was set as absence of nanoparticles. The cells were incubated at 37 °C, and the cell viability at different incubation times (12, 24, and 48 h) was analyzed. 100 μL of MTT reagent was added to each well followed by 2–4 h incubation at 37 °C temperature. Finally, after incubation, the media were replaced by 100 µL of dimethyl sulfoxide and well mixed. A microplate reader measured the absorbance at the wavelength of 570 nm. The relative cell viability percentage is evaluated by the following equation:

$$\mathrm{Cell}\;\mathrm{viability}\;\left(\%\right)=\frac{Absorbance\;of\;treated\;cells}{Absorbance\;of\;controlled\;cells}\times 100\%$$

#### 2.7.2. Trypan Blue Study

MG-63 cells were cultured (10^5^ cells/cm^2^) in the DMEM complete media at 37 °C. Synthesized IO, HAp, and IO-HAp nanoparticles were tested for in vitro cytotoxicity testing. The sample of 100 µg/mL concentration was mixed into the culture media. After 24 h, the cells were stained with trypan blue, and after washing with Phosphate buffer saline (PBS) the cells were examined under the microscope.

### 2.8. Nanoparticles Internalization Study by Cells

The MG-63 osteosarcoma cells were seeded at a cell density of 10^5^ cells/mL in a 35-mm culture plate and incubated in DMEM medium for 24 h. Only IO-HAp nanoparticles (100 µg/mL) were incubated with the attached MG-63 cells for 12 h. Following the incubation with the nanoparticles, the cells were stained with the 0.01% (*w*/*v*) proflavine solution. After incubation for 30 min, the dye was removed and washed gently with PBS solution to remove the nanoparticles’ excess and 100 µL of trypsin were added. After 2 min of incubation with trypsin, 100 µL of medium was added and finally observed under the fluorescence microscope (LEICA DMI 3000B, Wetzlar, Germany) with external magnetic force.

### 2.9. Magnetic Hyperthermia Experiment

The heating efficiency of the IO and IO-HAp nanoparticles was assessed in the presence of A/C magnetic hyperthermia device. The study was performed at 180 Gauss magnetic field strength with 409 kHz frequency. Approximately 100 µg/mL of the IO and IO-HAp nanoparticles were dispersed in water and were placed in a 6.5-cm radius copper coil.

### 2.10. In Vitro Magnetic Hyperthermia

To investigate the in vitro hyperthermia effects of IO and IO-HAp, nanoparticles MG-63 osteosarcoma cells were seeded at a cell density of 10^5^ cells/mL in a 35-mm culture plate with 2.5 mL of medium and incubated for 24 h. The cells with no nanoparticles were considered as the control and were incubated for 12 h. For the experimental study, cells were incubated for 12 h with 100 µg/mL of IO and IO-HAp nanoparticles. The experimental cell plates were positioned inside the hyperthermia coil and a 0.6 Tesla A/C magnetic field was applied with a frequency of 307 kHz and amplitude of 628 Oe for 30 min. The temperature was recorded with an optical thermocouple (AMOTH 8000, Anritsu Meter Co. Ltd., Tokyo, Japan), and was also observed under Infrared (IR) thermal camera.

## 3. Results and Discussion

### 3.1. Morphological Characterizations of the Materials

#### 3.1.1. X-ray Diffraction (XRD) Analysis

**Figure 2:**
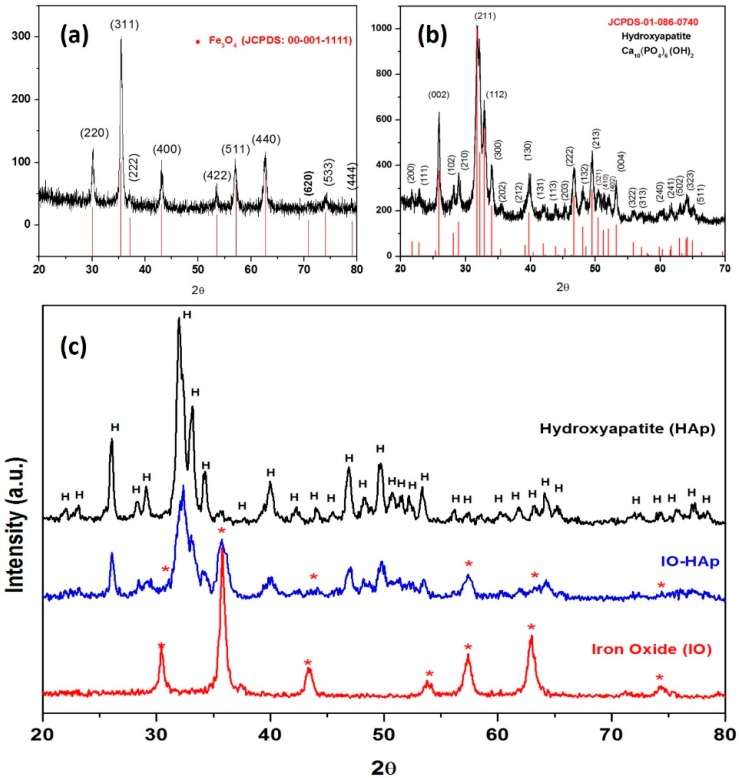
X-ray diffraction (XRD) analysis of (**a**) IO, (**b**) HAp and (**c**) comparing HAp, IO-HAp, and IO nanoparticles.

The sharp peaks for the calcined HAp and IO nanoparticles reveal their high crystallinity (Figure 2a,b). Well-resolved highest intensity peak for HAp was obtained at 2θ = 31.7° corresponding to (211) plane and for IO nanoparticles at 2θ = 35.5° for (311) plane. The presence of HAp characteristic peaks near 30°, 34.2°, 40°, 46°, 49°, and 53° confirms that the synthesized material as HAp. Along with the most prominent peak at (311) plane for Fe_3_O_4_ (IO) other associated peaks at 30.4°, 43.3°, 53.7°, 57.4°, 62.9°, and 74.2° are well resolved in XRD graph. In IO-HAp nanoparticles, XRD characterization, all of the HAp, and IO associated peaks are well observed (Figure 2c). Most of the peaks for IO and HAp are merged together in IO-HAp sample, but their increased intensity confirms their peak position in the synthesized IO-HAp nanomaterials.

#### 3.1.2. Fourier Transform Infrared Spectroscopy

**Figure 3:**
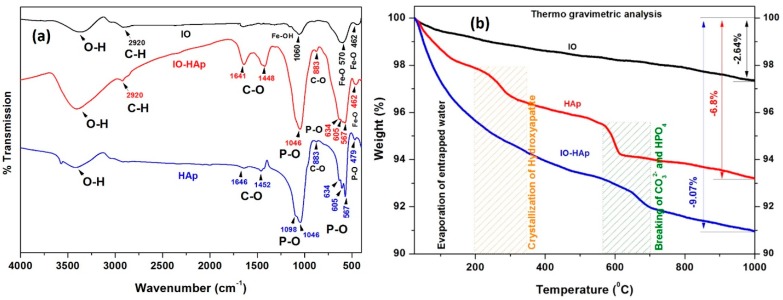
(**a**) FTIR and (**b**) Thermogravimetric analysis of IO, HAp and IO-HAp nanoparticles.

The FTIR analysis for pristine HAp, IO, and IO-HAp are shown in Figure 3a. The functional groups of the prepared samples confirm the presence of pristine IO and HAp. In IO-HAp nanoparticles, with additional peaks corresponding to HAp and IO are confirmed the close interaction between IO and HAp (Table 1). The FTIR spectrum of HAp nanoparticles confirms the characteristic bands for PO_4_^3−^ appear at 479, 567, 605, 1046, and 1098 cm^−1^ [25,26]. The trace at 479 cm^−1^ is attributed to the *ν*2 bending vibration of P-O and the bands at 1046 and 1098 cm^−1^ to the *ν*3 vibrations of PO_4_^3−^ groups [27]. The absorption bands rising at 567 cm^−1^ and 605 cm^−1^ can be recognized to the *n*4 bending mode of (PO_4_^3−^) functional group, and the peaks at 1098–1046 cm^−1^ represents the *n*3 vibrations of (PO_4_^3−^). The FTIR spectra demonstrate the absorption bands at 3450–3575 cm^−1^ and 634 cm^−1^ due to the stretching and libration modes, of the OH^−^ ions. The spectra of IO nanoparticles show a sharp band at 570 cm^−1^, which belongs to Fe–O vibrations for IO. The weak vibration monitored at 1060 cm^−1^ is characteristic of surface Fe–OH groups [28]. The FTIR spectra of IO-HAp nanoparticles show both the IO and HAp functional groups. The HAp functional groups in the range of 1098–1046 cm^−1^ have the higher intensities, which merges with the functional group of Fe–O in that region. Other important characteristic peaks are observed at 2790 cm^−1^ due to the presence of C–H groups [29]. This peak appears possibly due to the use of ethylene glycol during the synthesis of iron oxide nanoparticles.

#### 3.1.3. Thermogravimetric Analysis

The HAp, IO, and IO-HAp nanoparticles were studied for their thermal stability by thermogravimetric analysis in inert (nitrogen) atmosphere. The thermal decay curves (Figure 3b) show the differences in residual mass of the samples with controlled heating. The total weight loss from pure IO was approximately 2.64% during the experimental study due to the evaporation of adsorbed physical and chemical water. For HAp and IO-HAp nanoparticles weight losses were 6.8% and 9.07%, respectively. Moreover, for HAp and IO-HAp, the first weight loss was recorded at 80–200 °C due to entrapped physical water and at 200–500 °C temperature due to the crystallization of HAp nanoparticles, and the third weight loss that was related to the decomposition of remaining chemical complexes. At a higher temperature, no such major weight loss was detected. TG analysis of the IO-HAp nanoparticles exhibited weight loss of up to 850 °C temperature, and thereafter weight losses were negligible. The thermogravimetric study reveals the thermal stability of these nanoparticles with increased heating in a controlled atmosphere.

#### 3.1.4. FETEM, DLS and Energy Dispersive X-ray Spectroscopy (EDS) Analysis

**Figure 4:**
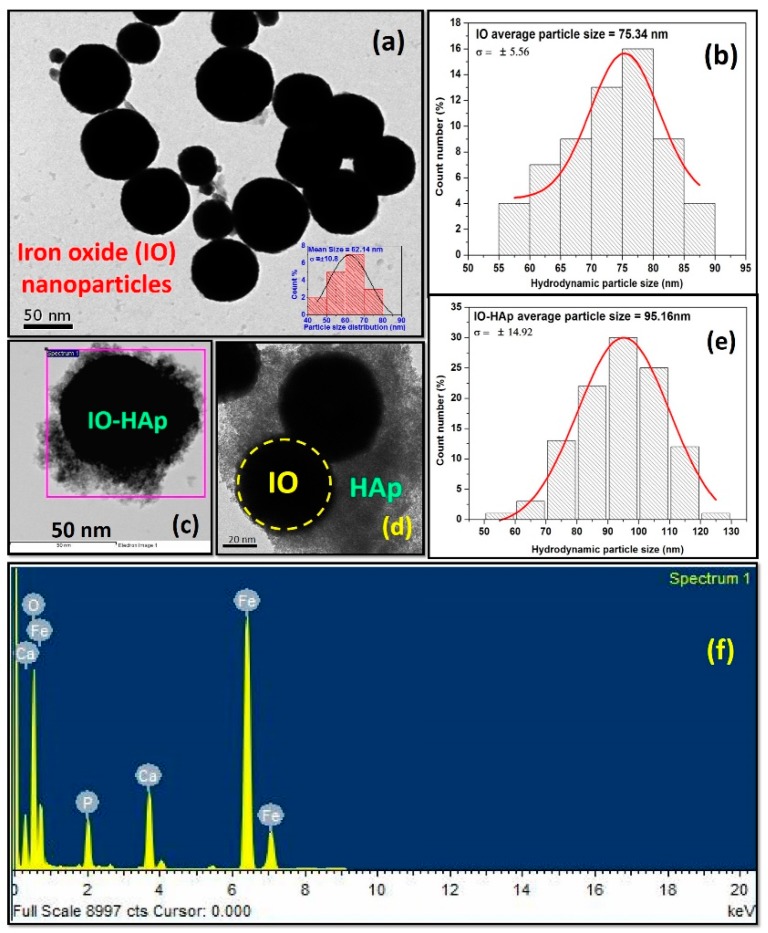
Field emission transmission electron microscopy (FETEM) analysis of (**a**) IO (inset particle size distribution) (**b**) dynamic light scattering (DLS) particle size distribution of IO nanoparticles, (**c**) and (**d**) FETEM analysis of IO-HAp nanoparticles (**e**) DLS particle size distribution of IO-HAp nanoparticles (**f**) EDS analysis of IO-HAp nanoparticles.

The FETEM study revealed that the synthesized IO nanoparticles are spherical in structure and well dispersed, as presented in Figure 4a. The particle size distribution of synthesized IO nanoparticles shows spherical morphology with an average size 62.14 ± 10.8 nm. The HAp nanoparticles coating over IO nanoparticles are shown in Figure 4c,d. The EDS analysis confirmed the presence of Fe, Ca, P, and O with Ca/P ratio of 1.70 (Figure 4f). The synthesized IO and IO-HAp nanoparticles average hydrodynamic particle size diameters were studied by laser mediated dynamic light scattering method (Figure 4b,e). The average size distribution of IO nanoparticles was determined as 75.34 ± 5.56 nm, and for IO-HAp was 95.16 ± 14.92 nm, which is very close resemblance with TEM calculated data.

#### 3.1.5. Vibrating Sample Magnetometer

**Figure 5:**
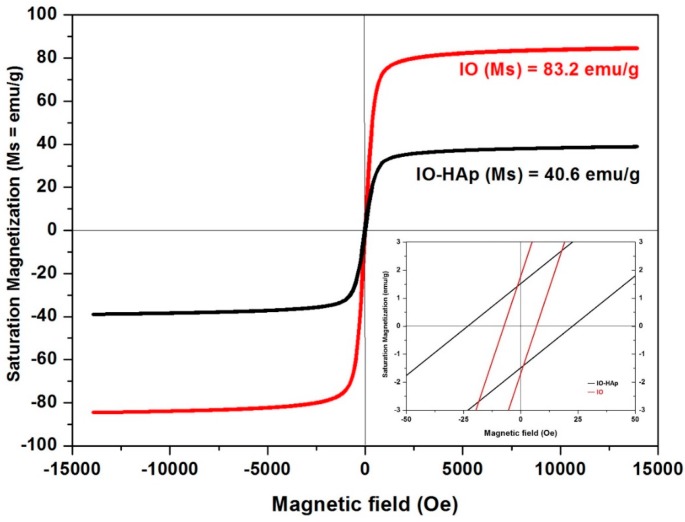
Magnetization curve of IO and IO-HAp nanoparticles (inset corresponds to a low-field region of IO and IO-HAp nanoparticles magnetic saturation curve to identify the coercivity and remanent magnetization).

The magnetic hysteresis loops for IO and IO-HAp nanoparticles are depicted in Figure 5. The IO nanoparticles show strong magnetic behavior, with the saturation magnetization of 83.2 emu/g; whereas, IO-HAp shows relatively low magnetic saturation of 40.6 emu/g, at 300 K and 1.5 T. The magnetic properties of HAp coated IO (IO-HAp) by different researchers compared with our present study are mentioned in Table 2. The saturation magnetization of pure IO is high (83.2 emu/g) whereas after coating the saturation decreases (40.6 emu/g) due to the coating of ceramic HAp. In spite of having comparatively low magnetic saturation, it is sufficient to treat for magnetic hyperthermia study, and also more than 20 emu/g magnetic saturation of IO-HAp nanoparticles are very few reported. Both IO and IO-HAp nanoparticles show the characteristic superparamagnetic behavior, with very less coercivity and remanent magnetization. The pristine HAp does not show any hysteresis loop in the magnetic field.

#### 3.1.6. Cytotoxicity Testing

The primary biocompatibility (in vitro cytotoxicity) screening is very important for materials that are used in the biomedical field. Ajeesh et al. reported that 40 wt % of Fe_3_O_4_-HAp does not harm osteoblast cell activity [16]. Sarath Chandra et al. reported cobalt- doped HAp nanoparticles as a promising material for drug delivery, magnetic imaging, and hyperthermia-mediated cancer treatment [17]. The cobalt co-doped HAp materials show good biocompatibility on blood cells (<5% hemolysis), with superior antimicrobial activity [18]. Hou et al. studied the in vivo hyperthermia effect of magnetic HAp nanoparticles to treat tumors. This cytotoxicity study that was conducted with blood samples from experimental mouse reveals good biocompatibility of magnetic HAp nanoparticles [22].

**Figure 6:**
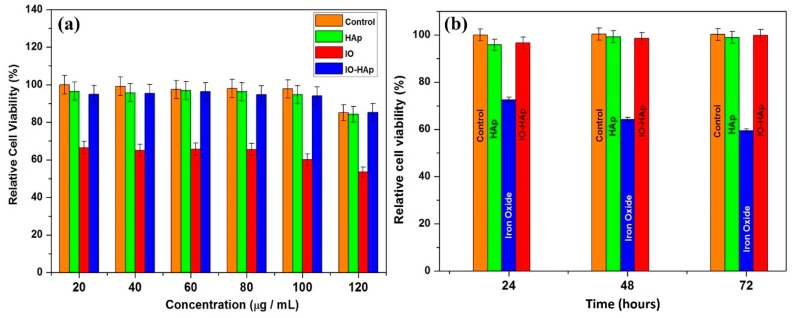
Cell viability study of MG-63 cells (**a**) incubated with different concentration HAp, IO, and IO-HAp nanoparticles for 24 h and (**b**) incubated (100 g/mL) nanoparticles with different time period (24, 48 and 72 h).

The MTT assay was performed for the HAp, IO, and IO-HAp nanomaterials using MG-63 (osteosarcoma) cells. Different concentrations (20, 40, 60, 80, 100, 120 µg/mL) of the HAp, IO, and the IO-HAp nanoparticles were studied for MTT assay. The relative percentage of cell viability is shown in Figure 6. The results show that the HAp and IO-HAp nanoparticles are cytocompatiable on MG-63 cells, whereas, IO nanoparticles shows marked toxicity. The relative percentage of cell viability suggests the maximum toxicity (approx. 42% cell death) that is shown by IO nanoparticles with the concentration of 120 g/mL. The MG-63 cells well tolerate the nanoparticles loading concentration up to 100 g/mL. The study was further performed by 100 g/mL concentration nanoparticles for 48 and 72 h (Figure 6b). The time duration study for cell cytotoxicity estimation confirms the maximum toxicity effect by IO nanoparticles, whereas HAp and IO-HAp nanoparticles show nontoxic behavior. As HAp is an excellent biocompatible material for this the coating of HAp over IO nanoparticles makes the composite nontoxic.

#### 3.1.7. Trypan Blue Study

**Figure 7:**
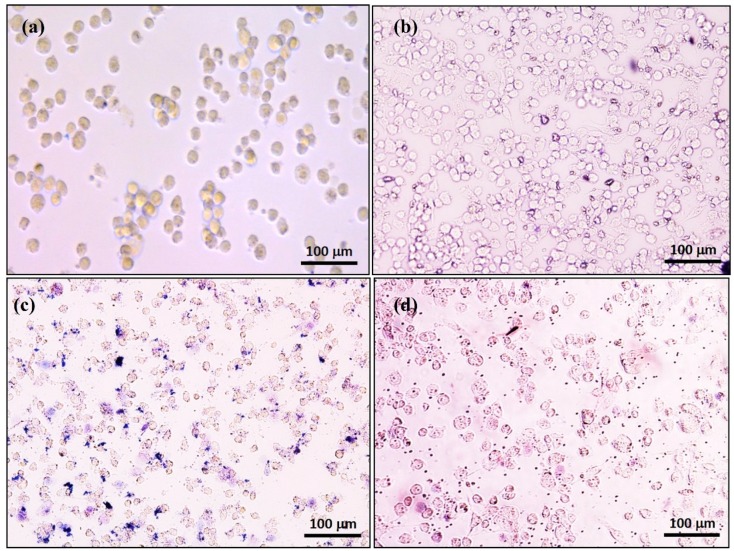
Trypan blue cell viability study of MG-63 cells incubated (**a**) without any nanoparticles as control (**b**) HAp, (**c**) IO, and (**d**) IO-HAp nanoparticles for 24 h with a concentration of 100 µg/mL.

The experimental results showed that almost all of the cells are healthy and survived in the presence of synthesized HAp and IO-HAp nanoparticles. But, for IO nanoparticles, the cells died and retained the trypan blue stain due to the toxic effect of IO nanoparticles. The control well (without any nanoparticles) shows maximum cell proliferation that is almost similar with HAp nanoparticles containing culture well. The observed cells were healthy, as depicted in Figure 7. After the in vitro hyperthermia study, the cells are treated with trypan blue, but nearly all of the cells died due to hyperthermia effect and retained the trypan blue stain.

#### 3.1.8. Nanoparticles Internalization Study by Cells

In the hyperthermia study, cells must uptake the magnetic nanoparticles inside so that the heating effect will able to more effectively to kill the target cells. In this study, only the IO-HAp nanoparticles were incubated with the MG-63 cells. Due to the HAp biomaterials coating the IO-HAp, nanoparticles do not show any toxic effect and are easily engulfed by the MG-63 cells. To confirm the IO-HAp internalization by MG-63 cells the IO-HAp treated cells are placed on a rotating magnetic field. By the effect of external magnetic force, only the IO-HAp internalized cells showed rotational movements (Video S1: Video of MG-63 IO-HAp NPs.).

### 3.2. Magnetic Hyperthermia Ability

#### 3.2.1. Magnetic Hyperthermia on MG-63 Cells

**Figure 8:**
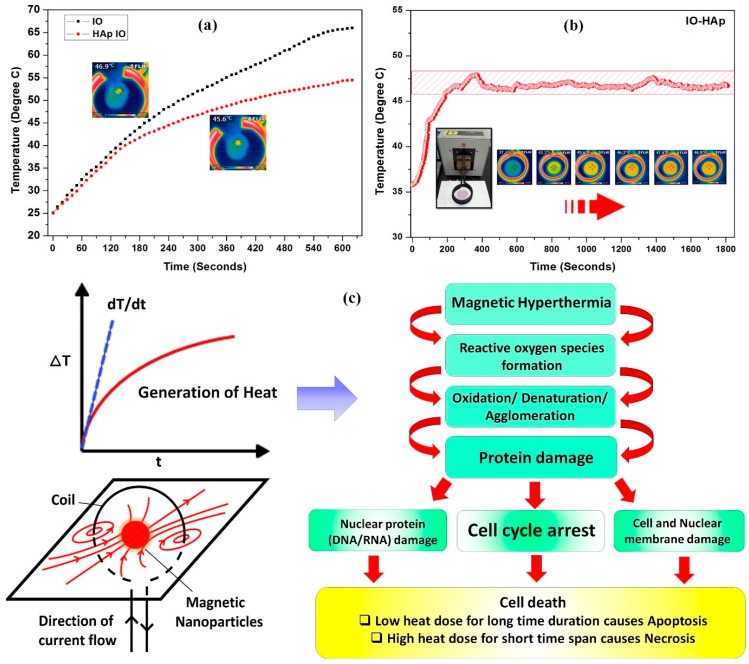
Magnetic hyperthermia study of (**a**) IO and HAp coated IO (IO-HAp) (**b**) hyperthermia study of IO-HAp and their corresponding infrared thermal images (**c**) hyperthermia and cell death mechanism.

Magnetic hyperthermia system could be used to kill cancer cells by their energy loss as heat (hysteresis loss). Only IO and IO-HAp nanoparticles are tested for the hyperthermia effect and for the specific absorption rate (SAR) value calculation. The synthesized IO and IO-HAp nanomaterials have the ability to produce heat in an alternating magnetic field. The hyperthermia experiment of IO and IO-HAp nanoparticles was initiated at 25 °C room temperature, and it reached 42 °C within 3 min after starting the experiment. The IO nanoparticles showed a very rapid temperature increase, and within 10 min it shows around 66 °C, however, IO-HAp reached a lesser temperature of about 54 °C (Figure 8a). The coating of HAp nanoparticles as an insulator over IO nanoparticles causes less of a temperature increase when compared to pristine IO nanoparticles. For efficient hyperthermia effect to kill the cancer cells, 42 °C to 47 °C temperature is ideal for a long time duration. The heating efficiency of the synthesized superparamagnetic IO-HAp nanomaterial was studied with MG-63 cells for 30 min incubation in hyperthermia field. When the temperature reaches to 47 °C, the hyperthermia instrument is controlled by power off to maintain the temperature between 42 °C and 47 °C for 30 min. All of the temperatures were measured by thermocouple and infrared thermal camera (Figure 8b).

The hyperthermia mediated cancer treatment is promising due to its less susceptibility to temperatures in the range of 42–47 °C, where normal cells are unaffected. The heating by magnetic particles due to the effect of magnetic field can occur via several mechanisms. The most common heating mechanism is due to the induced Eddy currents, which occur in bulk materials. The Eddy current for nano-sized materials heating is very weak because of the poor electrical conductivity of magnetic nanoparticles. Additionally, heating due to the hysteresis loss is dominant for this type of nanomaterials. Nanoparticles especially exhibit hysteresis loss due to Néelian and Brownian relaxation. The rotation of individual magnetic moments within the nanoparticles causes heat generation, referred to as the Néelian relaxation. In contrary, the Brownian relaxation mediated heat generation is caused due to the physical rotation of particles with the alignment process of magnetic moments (Figure 8c). After the hyperthermia treatment of MG-63 cells, trypan blue and TEM analysis were performed to analyze the cellular viability. In trypan blue assay, all of the cells retained the trypan blue stain, which confirms the effectiveness of nanoparticles in hyperthermia. In TEM analysis of treated MG-63 cells, the cells were found arrested in necrosis phase. This could be attributed to the effect of heat that is generated by magnetic hyperthermia reactive oxygen species (ROS). Gu et al., 2014, reported that the generation of ROS by heat stress might be involved with transcription-independent p53-mediated mitochondrial pathways [39]. Skibba et al., 1991, reported that hyperthermia mediated oxidative stress causes irreversible hepatocellular injury [40]. Additionally, the cellular internalization of nanoparticles could have induced the ROS, which causes oxidation and denaturation in cellular system results in damage of proteins, nucleotides, and membranes and finally resulting in cell death. In this study, the TEM analysis revealed that cell necrosis results in cell degradation and formation of blebs, which causes deformities or lysis of the cells (figure in Scheme 1).

#### 3.2.2. Measurement of SAR Effect

The heating efficiency of the nanoparticles in presence of A/C magnetic field was calculated as the specific absorption rate (SAR), and defined as the amount of energy transformed to heat per time and mass [41,42].

*SAR* is calculated by the initial linear temperature increase (Δ*T*) per time interval (Δ*t*).

(1) $$SAR(W/g)=\frac{c}{m}\times \frac{\Delta T}{\Delta t}$$

where, *c* (JL/K) denotes the water, specific heat capacity (4185 J L/K), and *m* (g/L) is the concentration of magnetic materials in water or liquid media.

The slope Δ*T*/Δ*t* curve was calculated up to first 60 s only (Figure 8c). The estimated *SAR* value for the IO and IO-HAp nanoparticles were calculated as 103 W/g and 85 W/g, respectively. The hyperthermia mediated heating effectiveness recommends the potential application of synthesized IO-HAp nanoparticles as nano heater for magnetic hyperthermia-mediated cancer therapy.

## 4. Conclusions

The synthesis and fabrication of magnetic nanomaterials with high biocompatibility are important for application in the biomedicine. HAp coated IO nanoparticles (IO-HAp) were fabricated as an effective nano heater for hyperthermia cancer therapy. Due to the unique surface activity of HAp, the composite nanoparticles did not require any further chemical modification. The synthesized composite IO-HAp nanoparticles were hydrophilic and superparamagnetic with good magnetic saturation of 40.6 emu/g. Without the magnetic field, the synthesized nanoparticles show minimal or no cytotoxic effect on cell lines. The cytotoxicity assay study revealed that the hyperthermia (~45 °C) mediated cell death to the cancer cells. The synthesized nanoparticles show an enhanced heating efficiency as nano heater when compared to conventional HAp coated iron oxide nanoparticles with a SAR value of 85 W/g when placed to A/C magnetic field. IO-HAp combined with magnetic hyperthermia can be a safe and effective therapeutic tool for different types of cancer treatment.

## Figures and Tables

**Table 1:** Fourier-transform infrared spectroscopy (FTIR) spectra analysis of HAp, IO and IO-HAp nanoparticles.

Sl. No.	Wavenumber cm^−1^			Functional Groups	Reference
	HAp	IO	IO-HAp		
1	3450–3575	3450–3575	3450–3575	O–H group stretching vibration	[25,26]
2	462–479	479	462	*ν*2 bending vibration of P–O	[27]
3	1646	--	1641	O–H adsorbed water	[30]
4	1452	--	1448	C–O stretching vibration	[30]
5	--	1060	--	weak vibration of Fe–OH group	[28]
6	1098–1046	--	1046	*n*3 vibration of P–O	[25,26]
7	883	--	883	*v*2 bending C–O	[30]
8	634	--	634	libration mode of the O–H	[25,26]
9	605	--	605	*n*4 bending mode of P–O	[25,26]
10	567	--	567	*ν*4 bending of P–O group	[25,26]
11	--	570	--	Fe–O vibrations for IO	[25,28]
12	--	2920	2920	C–H vibration	[29]

**Table 2:** Different synthetic routes application and their corresponding saturation magnetization (Ms) value of magnetic hydroxyapatite nanoparticles.

Sl. No.	Synthesis Route	Application	Saturation Magnetization (Ms)	Reference
1	Wet precipitation technique	Biomedicine application, Hyperthermia study.	7.23–20.92 emu/g	[22,31]
2	Pulsed plasma deposition	Biofilm formation	0.26 emu/g	[32]
3	Hydrothermal method	Biomedicine applications	~0.32 emu/g	[33]
4	Spray-drying technique	Medical diagnosis and imaging	~12 emu/g	[20]
5	Microwave route	pH-responsive drug release	18.9 emu/g	[34]
6	Multi step synthesis: wet precipitation, hydrothermal, ultrasonication, and layer by layer coating	Magnetic resonance imaging, Drug delivery	~4–7 emu/g	[35]
7	Hydrothermal method	pH dependent protein adsorption release carrier	11.5–15.5 emu/g	[36]
8	Polymer templated electrospun technique	Biomedical and hyperthermia treatment	27.20 emu/g	[37]
9	Ultrasonic irradiation technique	Biomedical	7.40 emu/g	[38]
10	Chemical precipitation	Magnetic hyperthermia for cancer treatment	40.6 emu/g	Present Study

## Supplementary Materials

The following are available online at www.mdpi.com/2079-4991/7/12/426/s1, Video S1: Video of MG-63 IO-HAp NPs.
